# The Use of BASILICA Technique to Prevent Coronary Obstruction in a TAVI-TAVI Procedure

**DOI:** 10.3390/jcm10235534

**Published:** 2021-11-26

**Authors:** Ana Paula Tagliari, Rodrigo Petersen Saadi, Eduardo Ferreira Medronha, Eduardo Keller Saadi

**Affiliations:** 1 Postgraduate Program in Cardiology and Cardiovascular Science Universidade Federal do Rio Grande do Sul, Porto Alegre 90035-002, Brazil; rodrigosaadi@terra.com.br (R.P.S.); esaadi@terra.com.br (E.K.S.); 2 Cardiac Surgery Department, Hospital São Lucas da PUC/RS, Porto Alegre 90619-900, Brazil; 3 Cardiac Surgery and Radiology Department, Hospital Mãe de Deus, Porto Alegre 90880-0481, Brazil; medronha@gmail.com; 4 Cardiac Surgery Department, Hospital de Clínicas de Porto Alegre, Porto Alegre 90035-903, Brazil

**Keywords:** transcatheter aortic valve implantation, transcatheter aortic valve replacement, BASILICA, coronary artery obstruction

## Abstract

Transcatheter aortic valve implantation (TAVI) to manage structural bioprosthetic valve deterioration has been successful in mitigating the risk of a redo cardiac surgery. However, TAVI-in-TAVI is a complex intervention, potentially associated with feared complications such as coronary artery obstruction. Coronary obstruction risk is especially high when the previously implanted prosthesis had supra-annular leaflets and/or the distance between the prosthesis and the coronary ostia is short. The BASILICA technique (bioprosthetic or native aortic scallop intentional laceration to prevent iatrogenic coronary artery obstruction) was developed to prevent coronary obstruction during native or valve-in-valve interventions but has now also been considered for TAVI-in-TAVI interventions. Despite its utility, the technique requires a not so widely available toolbox. Herein, we discuss the TAVI-in-TAVI BASILICA technique and how to perform it using more widely available tools, which could spread its use.

## 1. Introduction

The introduction of transcatheter aortic valve implantation (TAVI) in 2002, as an alternative to treat patients with severe aortic valve stenosis who previously had only surgery as an intervention option, represented a huge mark in the structural heart disease management revolution [1].

Recently, the American and European Guidelines for the management of valvular heart disease have recommended TAVI in several clinical scenarios provided that the anatomy is favorable for performing a transfemoral approach [2,3]. According to the American College of Cardiology (ACC) and American Heart Association (AHA) Guideline, TAVI may be considered in patients above 65 years and should be the first choice in those above 80 years [2]. To the European Society of Cardiology (ESC) and European Association for Cardio-Thoracic Surgery (EACTS) Guideline, TAVI should be chosen for those above 75 years and with high surgical risk (STS score or EuroScore ≥ 8) [3]. These changes in the last Guidelines, compared to the previous ones, were corroborated by important randomized clinical trials, whose results showed TAVI non-inferiority, or even superiority, compared to surgical aortic valve replacement (SAVR), in low-risk patients with a mean age of 73 and 74 years in the PARTNER 3 and EVOLUT Low-risk trials, respectively [4,5].

Taking into account the increasing number of low-risk patients undergoing TAVI and their long-life expectancy, one can assume that patients could outlive the bioprostheses’ expected durability. Consequently, the number of repeated transcatheter interventions following the first TAVI, the so-called TAVI-in-TAVI procedure, is also expected to increase [6].

Even though less invasive, TAVI-in-TAVI is more challenging and carries a higher complication risk, mainly coronary artery obstruction, than TAVI in a native valve. In an attempt to reduce the risk of coronary artery obstruction during native or valve-in-valve interventions, the BASILICA technique (bioprosthetic or native aortic scallop intentional laceration to prevent iatrogenic coronary artery obstruction) was conceptualized [7]. However, the BASILICA employment during TAVI-in-TAVI lacks evidence.

Herein, we provide an updated and comprehensive literature review focused on TAVI-in-TAVI BASILICA, and we illustrate this concept with a case report.

## 2. TAVI-in-TAVI

TAVI-in-TAVI is defined as a second transcatheter heart valve (THV) deployment within a previously implanted bioprosthesis because of suboptimal device position and/or function, during or after the procedure [8].

In 2007, Ruiz C. et al. reported the first TAVI-in-TAVI performed three years earlier. At the index procedure, a patient with severe aortic regurgitation and moderate aortic stenosis was submitted to a CoreValve (Medtronic Inc., Minneapolis, MN, USA) implant. Due to the presence of severe aortic regurgitation immediately after the implant, a second CoreValve was required. Based on the success of this case, the authors suggested that the concept and durability of the TAVI-in-TAVI started to be demonstrated [9].

Nowadays, a second valve implantation is applied in a broad spectrum of acute or chronic scenarios [10]. The most common TAVI-in-TAVI indications are:

(a) As a bail-out approach: in an acute setting, as a rescue strategy undertaken due to unsuccessful or suboptimal implantation.
(b) Late THV failure: due to late structural valve deterioration (stenosis, regurgitation, or mixed disease).
(c) A combination of structural and non-structural valve dysfunction: a combination of paravalvular regurgitation (PVL) and bioprosthesis failure, which could require a combined approach, such as PVL closure and a new prosthesis implantation.

Although TAVI-in-TAVI can offer immediate rescue management, avoiding open cardiac surgery and cardiopulmonary bypass, this is not without inherent complication risks.

In 2014, Witkowsky A. et al. reviewed 43 articles reporting TAVI-in-TAVI cases. In most of them, TAVI-in-TAVI was used as a rescue intervention to manage suboptimal bioprosthesis function. Aortic regurgitation was the main reason for a second bioprosthesis implantation, and prosthesis malposition was the main underlying cause of TAVI failure (81%). The reported TAVI-in-TAVI success rate varied from 90% to 100%, and the 30-day mortality rate was 0–14.3% [11]. While in the PARTNER trial multiple valve implantation was required in 1–2% (Cohort B: 1.1%; Cohort A: 2%), Vrachatis DA et al. [12] reported that in the CoreValve U.S. Pivotal Trial multiple valves were implanted in 3.5–4.5% (Extreme Risk Cohort: 3.5%; High-Risk Cohort: 4.1%) [13,14,15,16]. Similarly, Makkar R.R. et al. described that, among 2554 consecutive patients reviewed from the PARTNER cohorts A and B and accompanying registries, TAVI-in-TAVI was required in about 2.5%. In most cases (89%), it was performed intra-procedurally. On multivariable analysis, TAVI-in-TAVI was an independent predictor of 1-year mortality (Hazard ratio (HR) 2.68, 95% Confidence Interval (CI) 1.34–5.36; *p* = 0.0055). The authors highlighted that these early results, which were largely derived from rescue TAVI-in-TAVI, should not be extrapolated to future populations, such as elective TAVI-in-TAVI for degenerated bioprosthesis [17].

A more detailed description of the most recent and relevant TAVI-in-TAVI studies is presented in Table 1.

## 3. TAVI-in-TAVI Complications

Regarding TAVI-in-TAVI major concerns, bioprosthesis malpositioning and deformation, critical coronary flow obstruction, and residual transvalvular gradients are the three most relevant [24]. While coronary artery obstruction incidence is low (<1%) in native TAVI, this risk increases by 4 to 6 times (2.5–3.5%) in valve-in-valve intervention, and it has been associated with approximately 50% in-hospital mortality [24,25,26].

Coronary artery obstruction occurs when the THV displaces the underlying surgical or native aortic valve leaflets outward, obstructing the coronary ostia directly or by sequestering the sinus of Valsalva at the sinotubular junction (STJ) [27]. Consequently, patients with low coronary ostia and narrow sinus of Valsalva have a higher risk of coronary obstruction [28]. Komatsu I et al. stated that, based on the anatomical relationship of the aortic root to the coronary ostium, three types of coronary ostia and aortic valve complex size could be identified, as follow [28]:

(a) Type I: coronary ostium lies above the top of the deflected native or bioprosthetic aortic valve leaflet. In this case, the deflected leaflet will not be able to cover the flow to the coronary artery, even if the sinuses are extremely narrow.
(b) Type II: coronary ostium lies below the top of the deflected leaflet. In this case, the risk of coronary obstruction will depend on the capacity of the sinuses to accommodate the deflected leaflet. In type IIA, the sinus is wide and coronary obstruction will not occur. In type IIB, the sinus is effaced and coronary obstruction can happen after TAVI.
(c) Type III: implanted leaflets extend above the STJ when deflected, which is especially common in supra-annular THV. In type IIIA, both the sinuses and the STJ are wide and this condition may not be at risk for coronary obstruction. In type IIIB, either sinuses or STJ are narrow and coronary obstruction may occur. In type IIIC, non-effaced sinuses may obstruct the inflow to the coronaries if the leaflets can be deflected above the STJ level and positioned close to the aortic wall.

Therefore, anatomies at risk for coronary obstruction would include types IIB, IIIB, and IIIC, and these conditions may require coronary obstruction protection with the BASILICA technique. Coronary obstruction risk assessment also includes the VTC measurement (virtual THV to coronary ostium distance). In the case of a VTC less than 4 mm, the BASILICA technique should be considered. When the VTC is > 4 mm, the risk for STJ-inflow obstruction should be evaluated by analyzing STJ and commissures relationship. If the VTSTJ (virtual THV to STJ distance) is small, then the BASILICA should also be considered [28].

Alternative approaches to reduce the risk of coronary occlusion include coronary protection with a supportive coronary guidewire, undeployed balloon, chimney technique, or snorkel stents [7,29,30].

TAVI-in-TAVI on supra-annular devices is considered especially risky as the new THV tends to push the prior leaflets against the original frame that extends above the STJ, potentially blocking coronary blood flow and limiting catheter access [18].

Buzzatti N et al. stated that while after a native TAVI the coronary access can be maintained through the open-cell stent, after a TAVI-in-TAVI the stent frames of the two prostheses will overlap, and the new stent will push and spread the previous leaflets over the original stent, converting it into a “covered” stent up to the edge of the leaflets. Thus, stents frame overlaps and loss of free-flow may impair both coronary flow and cannulation. According to these authors, the anatomical and device-related factors predisposing to increased risk of impaired coronary access after TAVI-in-TAVI are: [31]

(a) STJ: represents the critical anatomical bottleneck regulating the access to the aortic root and coronary ostia; shorter and narrower STJ will leave less free space between the aortic wall and the edge of the “covered” old TAVI stent frame;
(b) Height of the leaflets of the original device: is the first determinant of the level below which the previous stent frame will not be crossable anymore after the implantation of a second device. Higher leaflets will more easily impinge on the STJ and impair catheter movement in the aortic root;
(c) Depth of device implantation: it will also modify the height of TAVI leaflets in respect to the aortic root, therefore possibly jeopardizing coronary access.

## 4. The BASILICA Technique

The BASILICA technique was first reported by Kan JM et al. in 2018. In this first report, the authors described that the procedure was performed on a compassionate basis in seven patients. Procedural success was achieved in all patients, with no hemodynamic compromise, no coronary obstruction, stroke, or any major complications [7].

BASILICA main objective is to intentionally lacerate the native or bioprosthetic leaflets to prevent critical coronary obstruction using catheter electrosurgery. Thus, BASILICA directly addresses the pathophysiology of coronary artery obstruction by lacerating the leaflet in front of a threatened coronary artery. After laceration, the sliced leaflet will splay and create a triangular space (“triangle of flow”) that may permit blood flow towards the sinus and from it to the coronary artery [32].

In 2020, Kitamura et al. evaluated the feasibility of the BASILICA technique in patients with high risk of coronary obstruction. In this study, BASILICA was feasible in 95% of the cases and resulted in effective prevention of coronary obstruction in 90% of them. Complication rates were low, with no cases of major vascular complication, need for mechanical circulatory support, stroke, or mortality at 30 days. These results provide further evidence on the feasibility, efficacy, and relative safety of the BASILICA technique [33].

Westermann D et al. assessed BASILICA clinical outcome in a single-center cohort described as the Hamburg BASILICA experience. In this study, 15 consecutive high-surgical risk patients were enrolled and submitted to TAVI due to degeneration of stented (80.0%) or stentless (6.7%) bioprosthetic aortic valves, or native aortic stenosis (13.3%). Procedure feasibility was 86.7%, with no 30-day all-cause deaths or stroke [34].

In this same line, Tagliari et al. had described six cases of valve-in-valve BASILICA procedures. Median left and right coronary artery heights were 9.1 mm (6.2–10.3) and 12.4 mm (10–13.5), respectively, with a median VTC of 2.9 mm on the left and 4.6 mm on the right side. The success rate was 87.5%, and there were no intraprocedural complications, coronary obstruction, in-hospital death, valve complication, cardiovascular event, or pacemaker implantation [35].

Recently, the 1-year outcomes from the BASILICA trial were published. This study enrolled 30 patients (43% native and 57% bioprosthesis). The 30-day success rate was 93.3%, with a stroke rate of 10%, and 1 death. Between 30 days and 1 year, there were no additional strokes, no myocardial infarction, and two deaths (10% 1-year mortality). No patient needed repeat intervention for aortic valve or coronary disease. Despite these encouraging outcomes, the authors concluded that the “applicability of BASILICA for failed THV is potentially large, but early benchtop studies suggest that it may not be suitable in all TAVI-in-TAVI procedures because of THV design and randomness of commissural alignment” [36].

Investigating TAVI-in-TAVI BASILICA feasibility in a benchtop model, Khan JM et al. analyzed if leaflets from the four commonest THV (Evolut R, SAPIEN XT, SAPIEN 3, and Lotus) could be split longitudinally to mimic BASILICA laceration. After some tests, they observed that effective leaflet splay could be achieved in the older generation SAPIEN XT and Lotus valves, but the newer generation SAPIEN 3 and Evolut appeared to demonstrate less effective leaflet splay. The authors also commented that, even in the case of feasible BASILICA laceration, the new TAVI commissures might randomly align unfavorably and obstruct the splayed leaflet. Besides, if the new TAVI skirt is positioned too high, this might also obstruct the lacerated leaflet. Therefore, success or failure would depend on commissural alignment and depth of new TAVI device implantation [37].

There are several unique concerns when a TAVI-in-TAVI BASILICA is planned, such as to ensure that the guidewire does not traverse through the stent frame and stays within the previous THV and to avoid interaction of the wire loop with the lower skirt of the THV [38]. Another concern is the possibility that the outer TAVI leaflets could get pinned against their frame by the inner TAVI device and, thereby, failing to splay and allow coronary perfusion [27].

## 5. BASILICA Required Equipment

As described by Komatsu I et al. there are several not so commonly utilized equipment required to perform a BASILICA procedure, comprising a Snare system (Amplatz Gooseneck™), a 6 Fr multipurpose (MP) guide catheter, an Astato XS 20 300 cm guidewire (Asahi Intecc USA, Inc., Tustin, CA, USA), a PiggyBack^®^ Wire Converter (Vascular Solutions, Minneapolis, MN, USA), an 8 Fr guide catheter (8 Fr AL3 or AL1/2/4 or EBU 3.5/4 for left cusp; 8 Fr MP or JR for right cusp), a 125 cm diagnostic 5 Fr internal mammary (IMA) catheter, an electrosurgical generator, surgical pencil, ground pad, scalpel blade, and mosquito clamps. For a rapid new THV deployment after leaflet laceration, a pigtail positioned in the left ventricle, inserted in parallel to the traversal guide, is also recommended. The snare size is determined by the perimeter-derived diameter of the LVOT at 5–10 mm below the annulus plane [28].

Even though this toolbox is highly recommended, it is not available in many countries and centers, precluding a widespread BASILICA employment. Searching solutions and similar equipment to replace the traditional ones, we describe the case report below. This case also corroborates TAVI-in-TAVI BASILICA feasibility since, to the best of our knowledge, it is the second case report describing a TAVI-in-TAVI BASILICA.

## 6. Case Report

### 6.1. History of Presentation

An 86-year-old woman was admitted to a tertiary hospital with severe refractory heart failure secondary to severe aortic stenosis and moderate aortic regurgitation (New York Heart Association functional class IV). The patient was stable up to two years ago when she became lost to follow up.

Her previous medical history included arterial hypertension, persistent atrial fibrillation on oral anticoagulant therapy (rivaroxaban 10 mg/day), previous smoking, chronic obstructive pulmonary disease, previous breast cancer, colonic angiodysplasia, and diverticular disease. Regarding previous cardiac interventions, the patients had received a permanent pacemaker 1 year before due to tachycardia-bradycardia syndrome and a TAVI (23 mm CoreValve) in 2012 to treat severe aortic stenosis. STS score was 8% and EuroScore II 14.9%.

### 6.2. Preoperative Investigation

**Figure 1:**
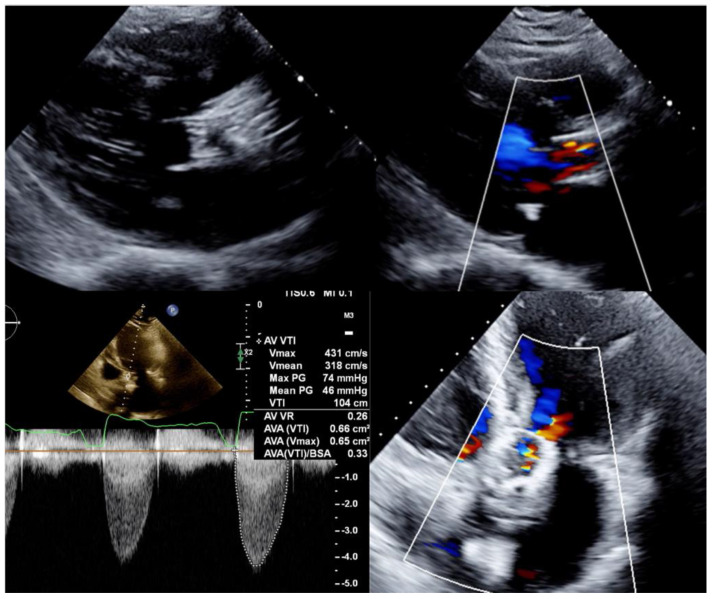
Transthoracic echocardiogram long-axis and 4-chamber views showing a degenerated CoreValve bioprosthesis with thickened leaflets, severe aortic stenosis, and moderate aortic regurgitation.

Transthoracic echocardiogram (Figure 1) showed moderate aortic valve regurgitation and severe aortic stenosis. Aortic valve peak and mean gradients were 74 mmHg and 46 mmHg, respectively, with an effective orifice area of 0.7 cm^2^ and a peak velocity of 4.3 m/s. Left ventricle ejection fraction was preserved (67%). In view of these findings, the diagnosis of structural bioprosthetic valve deterioration with severe BVF was stablished. Considering her high surgical risk and frailty condition, the heart team indicated a new transcatheter intervention (TAVI-in-TAVI).

**Figure 2:**
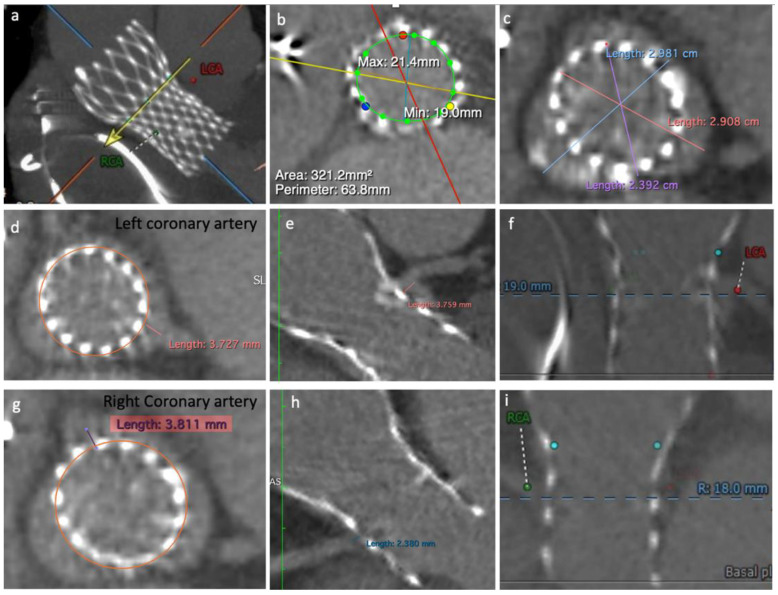
Computed tomography angiography images: (**a**) CoreValve structure, left coronary artery ostium (LCA) and right coronary artery ostium (RCA); (**b**) Calculated area and perimeter; (**c**) Sinus of Valsalva diameter; (**d**,**e**) left coronary cusp VTC; (**f**) LCA height related to the frame bottom; (**g**,**h**) right coronary cusp VTC; (**i**) RCA height related to the frame bottom.

A computed tomography angiography (CT) showed a degenerated 23 mm CoreValve bioprosthesis and almost immobile leaflets. Both coronary arteries Ostia were originated below the top of the CoreValve leaflets’ heigh: CoreValve leaflets’ height = 26 mm; left coronary ostium height (from frame bottom to coronary ostium) = 19 mm; right coronary artery ostium height = 18 mm. The calculated VTC was around 3.8 mm on both sides. Therefore, both coronary arteries were at risk of sinus sequestration and flow obstruction (Figure 2).

Since on the right side, a combination of low coronary artery height and too narrow sinus of Valsalva was observed, a condition described as a relative contraindication to BASILICA (the skirt of a new THV itself could potentially occlude the newly formed “triangle of flow”), we decided to protect the right coronary with a supportive coronary guidewire and an undeployed balloon, and proceed with the BASILICA in the left leaflet.

Searching for previous TAVI-in-TAVI BASILICA cases, none but one was found. In that case, the patient was at risk for sinus sequestration and impeding coronary access; thus, a left cusp BASILICA followed by a SAPIEN 3 implantation within a degenerated 31 mm CoreValve was performed. Regarding equipment, the authors described having used standard BASILICA equipment [38].

### 6.3. Procedure

Under general anesthesia, transesophageal echocardiography (TEE) guidance and full systemic heparinization, we accessed the right radial artery as a route to insert a protective guidewire in the right coronary artery. A temporary pacemaker was inserted in the right ventricle through the right femoral venous access.

After these steps, both right and left femoral arteries were punctured. The right one was used as the main access (14 Fr sheath), while the left was the contralateral access (7 Fr sheath). Through the right side, a 5 Fr pigtail was positioned in the left ventricle aiming to allow a fast new THV deployment if hemodynamic instability occurred after leaflet laceration.

**Figure 3:**
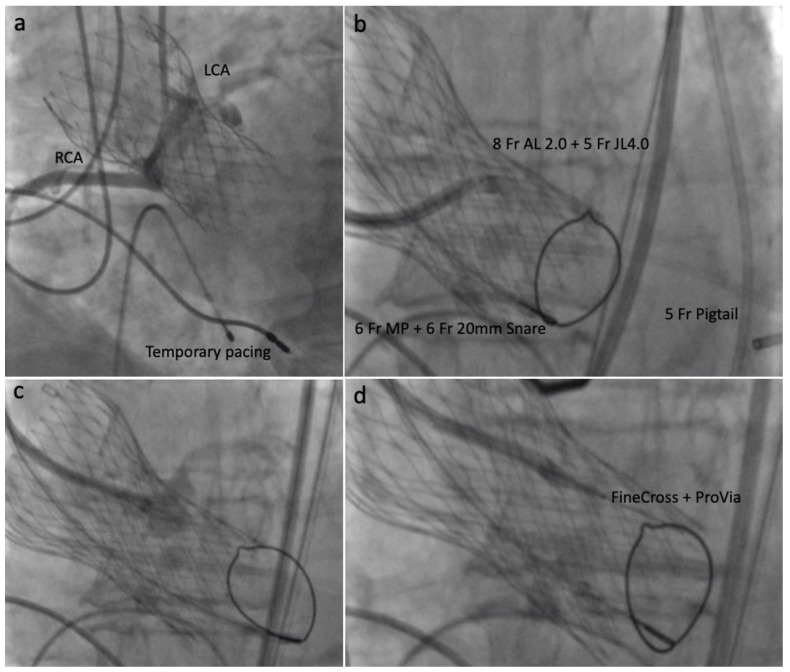
Intraprocedural steps: (**a**) Contrast injection showing right and left coronary ostia; (**b**) 8 Fr AL 2 catheter and 5 Fr × 125 cm JL 4.0 directed to the left leaflet mid base; (**c**) Contrast injection confirming proper position; (**d**) 0.014 × 300 cm ProVia guidewire and 1.8/2.6 Fr × 150 cm FineCross micro-catheter insertion.

Through the contralateral access, a 6 Fr 20-mm snare (ONE Snare, Merit Medical Systems) inside a 6 Fr MP guiding catheter (Medtronic) was positioned in the LVOT, 5–10 mm below the CoreValve. Through the main access, an 8 Fr AL 2 catheter (guide catheter) with an extra-long 5 Fr × 125 cm JL 4.0 catheter (child catheter) was positioned directed to the left cusp mid base. Replacing the Astato and the PiggyBack^®^ we used a 0.014 × 300 cm ProVia guidewire (Medtronic) insulated in a micro-guide catheter (FineCross MG 1.8/2.4 Fr × 150 cm, Terumo) (Figure 3).

**Figure 4:**
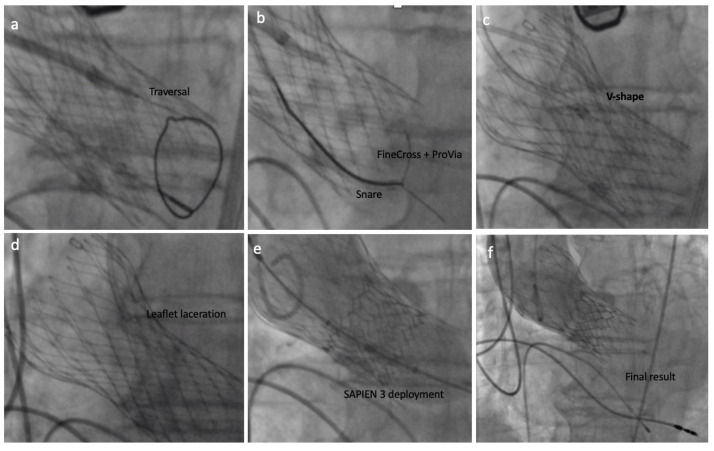
The BASILICA technique: (**a**) left coronary leaflet traversal; (**b**) guidewire snaring; (**c**) V-shape formation and delivery; (**d**) left coronary leaflet laceration; (**e**) SAPIEN 3 deployment; (**f**) final result assessment.

**Figure 5:**
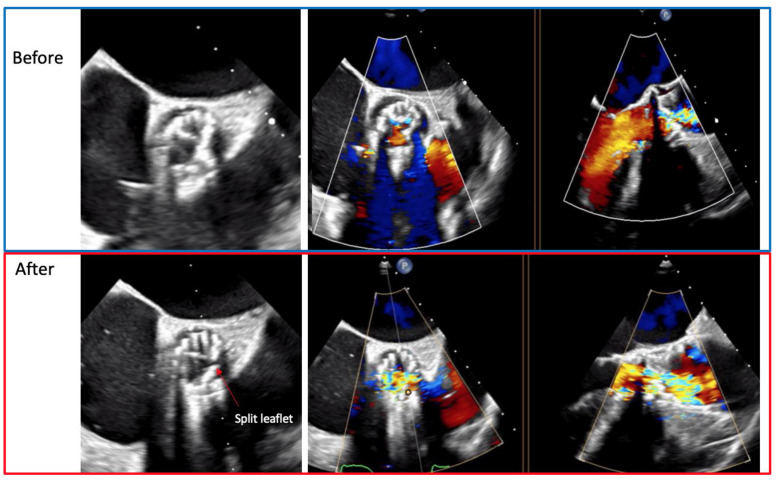
Transesophageal echocardiogram showing the left coronary leaflet before and after the laceration.

After fluoroscopy and TEE had confirmed the proper position, the back of the ProVia guidewire was scraped and connected with an electrical pencil. The electrosurgical generator was set on 70 W “pure cut” mode for traversal. Electricity was applied, and the leaflet traversed by the guidewire, which was snared. Once snaring was achieved, a V-shape was performed in the middle part of the wire by denuding it approximately 10 mm in its inner curve. By pulling the snare, the V-shape was advanced until the traversed point. At this point, simultaneous 5% dextrose was injected into each guide catheter and leaflet laceration was performed using 100 W power. Successful leaflet laceration was confirmed and BASILICA equipment removed. The remaining steps for complete a 23 mm SAPIEN 3 valve deployment were performed in a standard fashion (Figure 4 and Figure 5).

Final results showed proper SAPIEN 3 position, no PVL, no transvalvular aortic regurgitation, adequate gradients and coronary artery perfusion (Supplementary Figure S1). The patient remained stable during the whole procedure and presented no ECG change.

A summary of procedural steps and equipment used are presented in (Supplementary Table S1).

## 7. Discussion

In the last decades, the scientific community has seen a spreading application of transcatheter solutions to treat several structural heart valve diseases. Considering the rapid increase in the number of TAVI procedures, the need for subsequent reinterventions is expected to rise dramatically. In this setting, coronary artery access and coronary obstruction prevention become extremely relevant [6].

In order to facilitate future coronary access, approaches to achieve new THV commissural alignment have been recently described by Tang GHL and Tagliari AP et al. [39,40]. As we have previously described for the PORTICO platform, commissural alignment concept consists in finding a fluoroscopic projection where two native commissures are overlapped leaving the other one isolated. In a cusp overlap projection (RAO/CAUDAL), for instance, we know that two native commissures will be overlapped in the outer aorta curvature while the other one will remain isolated in the inner curvature. To achieve commissural alignment, we rotate the delivery system when it arrives in the descending aorta until the neo-commissures are displayed in the same way (two neo-commissures in the lateral aspect of the descending aorta and one isolated in the medial aspect of the descending aorta) [40]. Tang GHL et al. have suggested that with the EVOLUT platform a better commissural alignment can be achieved if we implant the delivery system with the flush port rotated from a 12 o’clock position to a 3 o’clock position [39].

It is relevant to highlight that during SAVR, commissural alignment is routinely achieved since native leaflets are resected, and surgeons align the commissural posts of bioprosthetic valves to native commissures to avoid coronary obstruction. However, SAVR following TAVI is an extremely risky procedure and with scarce data from large cohorts. Due to adhesions of the valve to the surrounding tissue, removing a THV poses a high risk, because it may disrupt the aortic root. Ando T et al. reported an in-hospital mortality for redo interventions of 7.6% (5.3% for redo TAVI or balloon valvuloplasty vs. 13.8% for redo SAVR, unadjusted *p* =0.10). Stroke, myocardial infarction, bleeding requiring transfusion, new pacemaker, and acute kidney injury rates were 4.7%, 2.6%, 9.3%, 10.0%, and 31.2%, respectively [41]. In this same line, Jawitz OK et al. pointed out that SAVR after a failure TAVI is a complex, technically demanding procedure, associated with long operative times, increased perioperative morbidity, and much higher than expected operative mortality when compared to redo SAVR. In this study, the authors included 123 patients (median age 77 years) from STS adult cardiac surgery database who underwent SAVR following TAVI between 2011 and 2015. The operative mortality rate was 17.1%, and the observed versus expected mortality ratios were heightened regardless of baseline mortality risk (low 5.48; intermediate 1.66; high 1.16) [42].

Alternatives for the treatment of acute coronary occlusion following TAVI include snaring and removal of the THV or referral for urgent surgery. The employment of chimney stenting is a more reproducible and straightforward approach, whose results were recently published by Mercanti F et al. In the Chimney Registry, 60 cases were examined. Procedural and in-hospital death occurred in three patients. During a median follow-up of 612 days (405–842 days), 2 cases of stent failure were reported (1 in-stent restenosis, 1 possible late stent thrombosis). Discussing these results, authors commented that the BASILICA technique has advantages over chimney stenting, including the avoidance of placing a coronary stent in the aorta and the consequent risk for reaccessing coronaries, restenosis, and thrombosis. Familiarity with both, BASILICA and chimney stenting, is advised for TAVI operators. However, the efficacy of chimney stenting relative to an alternative management strategy, such as BASILICA or elective deferral to conventional SAVR, is unknown [43].

Here we provided a comprehensive review of TAVI-in-TAVI and BASILICA technique employment, outcomes, and concerns, adding evidence to support the technique feasibility and effectiveness.

We reported a Sapien 3 valve implantation inside a degenerated CoreValve bioprosthesis performed together with the BASILICA technique in a patient with high-risk of coronary obstruction. BASILICA was employed to lacerate the left coronary leaflet using not previously described alternative equipment. This report contributes to supporting TAVI-in-TAVI BASILICA’s feasibility and safety as a treatment option in patients at risk for coronary obstruction or sinus sequestration. Despite being just a case report, our article is the second one to report a successful TAVI-in-TAVI BASILICA.

There is no doubt that TAVI-in-TAVI BASILICA is an extremely complex and risky procedure, with a high chance of non-success due to several factors. However, when we face highly complex patients, with contraindication to open cardiac surgery, we need to find alternative solutions and push our limits. As said by Vavuranakis M et al. “various technical issues and complications urged pioneer “structuralists” to discover solutions” [44].

## 8. Conclusions

TAVI-in-TAVI is a growing field that offers a less invasive alternative to treat degenerated THV. However, the inherent TAVI-in-TAVI procedural risks, especially coronary artery obstruction, should be considered. Careful preprocedural planning and an integrated heart team approach are essential to a successful TAVI-in-TAVI procedure. TAVI-in-TAVI BASILICA is a promising new transcatheter solution but needs further studies to be validated.

## Figures and Tables

**Table 1:** Most recent and relevant TAVI-in-TAVI studies.

Author and Year	Number of Patients	Recruitment	Follow-Up	Survival at 30 Days and 1 Year	Device Success **
Percy, ED. 2021 [18]	617	All Medicare beneficiaries who underwent TAVI from 2012 to 2017	1 year	94% at 30 days and 78% at 1 year	----
Attizzani, GF. 2021 [19]	292	All TVT Registry patients who underwent redo-TAVI with Evolut platform between April 2015 and March 2020	1 year	96.8% at 30 days and 82.3% at 1 year	94.5%
Landes, U. 2020 [20]	212	Redo-TAVI registry, 37 centers	30 days	94.6% and 98.5% for early and late valve dysfunction *	85.1%
Toggweiler, S. 2012 [21]	21	Three Canadian centers, between January 2005 and March 2011	1 year	85.7% at 30 days and 76% at 1 year	90%
Schmidt, T. 2016 [22]	19	Consecutive patients in 2 German centers, between October 2011 and November 2015	1 year	89% at 30 days and 67% at 1 year	89%
Tsuda, M. 2019 [23]	6	Osaka University Hospital, between October 2009 and June 2018	1 year	100% at 30 days and 83.3% at 1 year	83.3%

* The study considered early valve dysfunction when it occurred within the first year after first valve implantation, and late if after one year. ** According to VARC-2 criteria.

## Supplementary Materials

The following are available online at https://www.mdpi.com/article/10.3390/jcm10235534/s1. Figure S1: Transesophageal echocardiogram short and long axis views showing: (a) new implanted SAPIEN 3 with no residual aortic regurgitation or aortic stenosis; (b) color Doppler images. Table S1: TAVI-in-TAVI BASILICA equipment.
