# Supplementary material for: The Use of BASILICA Technique to Prevent Coronary Obstruction in a TAVI-TAVI Procedure
Source: J Clin Med. 2021 Nov 26;10(23):5534. doi: 10.3390/jcm10235534 (PMC8658292; doi:10.3390/jcm10235534)
Supplement: Supplementary file 1 [file jcm-10-05534-s001.zip › jcm-1409352-SI.pdf]

The use of BASILICA technique to prevent coronary obstruction in a TAVI-TAVI procedure

Supplementary File

Supplementary Figure S1. Transesophageal echocardiogram short and long axis views showing: (**a**) new implanted SAPIEN 3 with no residual aortic regurgitation or aortic stenosis; (**b**) Color Doppler images.

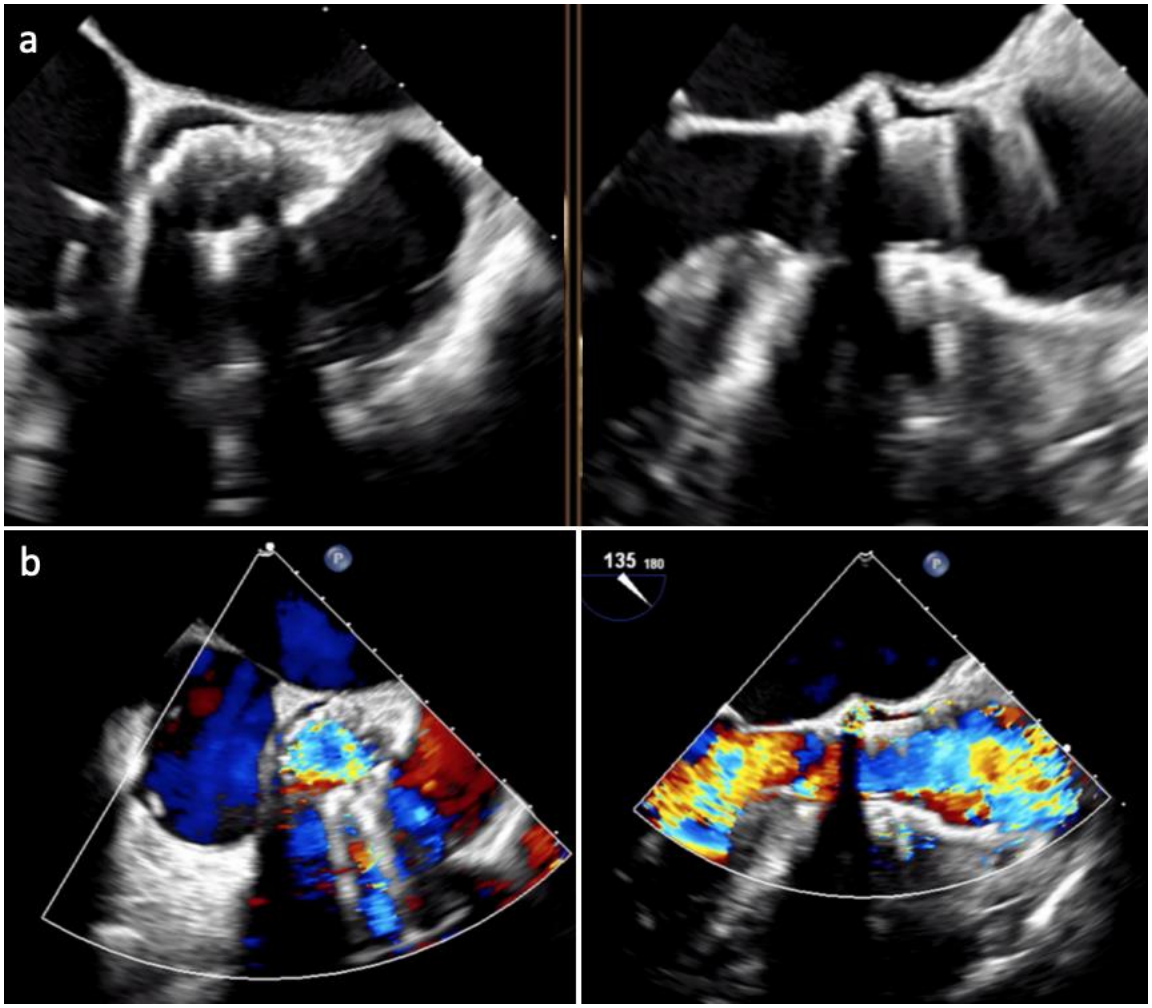

Supplementary Table S1. TAVI-in-TAVI BASILICA equipment.

Stage	Device
Access	Femoral closure device (Perclose Proglide Abbott).<br><br>Sheath:<br>-DrySeal 14-26Fr x 33cm (W.L. Gore & Associates)<br>Alternative: 14-16Fr femoral sheath (Cook) or<br>14-16Fr eSheath (Edwards Lifesciences)
Targeting and transversal	Snare System: 20-30mm Amplatz Gooseneck (Medtronic)

	Alternative: 6Fr 20mm ONE Snare (Merit Medical Systems)<br><br>Snare guiding cateter: 6Fr x 100cm MP or 6Fr x 100cm JR 3.5<br><br>For left leaflet BASILICA<br>Guide catheter: 8Fr x 100cm AL 1.0, AL 2.0, AL 3.0 or 7Fr EBU 3.75, EBU 4.0<br><br>Child cathteter: 5Fr x 125cm IM diagnostic catheter<br>Alternative: 5Fr x 125cm pigtail or 5Fr x 125 cm JR 3.5 or JL 4.0<br><br>Microcatheter: PiggyBack® Wire Converter (Vascular Solutions, Minneapolis, MN, USA)<br>Alternative: 1.8/2.4Fr x 150cm micro-guide catheter FineCross MG (Terumo)<br><br>Guidewire: 0.014 x 300cm Astato XS 20 (Asahi Intecc USA, Inc., Tustin, CA, USA)<br>Alternative: 0.014 x 300cm ProVia guidewire (Medtronic)
Laceration	Electrosurgical generator, surgical pencil and ground pad<br>Scalpel balde (nº 11)<br>Mosquito clamps
